# Development of microencapsulated anthocyanin-rich powder using soy protein isolate, jackfruit seed starch and an emulsifier (NBRE-15) as encapsulating materials

**DOI:** 10.1038/s41598-020-67191-3

**Published:** 2020-06-23

**Authors:** Avinash Singh Patel, Abhijit Kar, Debabandya Mohapatra

**Affiliations:** 1 Division of Food Science & Postharvest Technology, ICAR - Indian Agricultural Research Institute, New Delhi, 110012 India; 2 Department of Food Science and Human Nutrition, School of Food and Agriculture, University of Maine, Orono, ME 04469 United States; 3 Agricultural Produce Processing Division, ICAR-Central Institute of Agricultural Engineering, Bhopal, 462038 India

**Keywords:** Drug delivery, Drug development

## Abstract

A trend of present encapsulation research indicates an increased interest in the search for natural encapsulants for bioactive phytochemicals. The present study in pursuit of the same studies the use of jackfruit seed starch (JSS), an underutilized natural polysaccharide in conjugation with soy protein isolate (SPI) as an encapsulating material and NBRE-15 as an emulsifier. Three independent variables viz., total soluble solids (TSS, 20, 25 and 30° Brix), SPI: JSS (1:1, 1:3 and 1:5) and NBRE-15 (0.1, 0.2 and 0.3%) were optimized for achieving the most efficient encapsulation of anthocyanin using a three level, three parameter, Box-Behnken design (BBD) of the Design of Experiments (DOE). The responses considered for the optimization were monomeric anthocyanin content, antioxidant activity and encapsulation efficiency. A combination of 27.0% TSS, 1:5 SPI: JSS ratio and 0.3% NBRE-15 was found to be optimum for the encapsulation of anthocyanin with the desirability of 92.6%. Microcapsules obtained using the optimized combination of independent variables was found to contain 3215.59 mg/100 g monomeric anthocyanin. The antioxidant activity and encapsulation efficiency of the encapsulated material obtained using optimized combinations of independent variable were found to be 365.26 µmol Trolox/g and 89.71%, respectively. The microcapsules were also additionally analyzed for the particle size distribution and morphological characterization. Particle size analysis indicated that the microcapsules obtained had a mean particle size of 60.97 µm. Scanning electron microscopy for morphological characterization indicated that the microcapsules so obtained were oval to round in shape and had a smooth surface. Storage studies to estimate the half-life of anthocyanin in the microcapsule at room temperature (37 °C) clearly indicated greater stability i.e. 63 days when stored under amber-colored vial compared to only 35 days when stored under clear transparent vial.

## Introduction

Anthocyanins are water-soluble glycosides of anthocyanidins (aglycons), which are responsible for the color of petals, pericarps and other plant parts and contain A-, B-, and C-ring moieties with complex patterns of hydroxylation, methoxylation, glycosylation and acylation^1,2^. Anthocyanins are of major importance, due to their cardio-protective, anti-carcinogenic, anti-neurodegenerative, vision-improving and diabetes-preventing properties^3,4^. The color of anthocyanins have been reported to advance through red, purple and blue to green as pH increases^5^ and thermally stable below pH 3 and unstable at neutral pH^6^. Anthocyanins occur in foods rich in blue, purple, red or black in color such as blueberry, raspberry, black rice, black bean, black carrot and others^7–10^.

Black carrots (*Daucus carota* ssp. sativus) have their origin from Turkey, Middle East and Far East, where they have been cultivated for at least 3000 years^11^ and are a rich source of anthocyanins (1750 mg/kg)^12^. Black carrot juice has been reported to contain novel anthocyanins with unique aglycon moieties as a C4-substituted anthocyanin containing an additional pyran ring between C4 and the hydroxyl group at C5^13^.

Food industries are more and more interested towards the development of functional ingredients considering the increased demand by the consumer today for their food to have therapeutic value in addition to their intended/committed nutritional contents^14–18^. This had led to the demand for more and more functional foods which are expected to have nutraceutical properties. However, considering the fact that most of the functional/nutraceutical ingredients when extracted from their natural matrices are not stable and are mostly lost during the processing operations, the industry is more and more interested in looking for techniques and ingredients to stabilize these functional/nutraceutical ingredients before they can be incorporated into their intended food matrices. Encapsulation is one such process which denotes coating/covering of any targeted material (core) within microcapsules/microsphere (host) to protect it from the adverse environmental conditions (high acidity, oxygen stress, moisture and even gastric conditions in stomach and gastrointestinal tract), in addition to facilitating controlled release and targeted delivery^19–22^. These microcapsules/ microspheres are selected for their specific application depending upon the properties of core materials and the process of encapsulation^23–25^. The microcapsules/microsphere are expected to have low solubility, should be biodegradable, biocompatible and non-reactiveso that it is protected while it passes through *in vitro* and *in vivo* environmental conditions^22,26,27^. Composition of the coating material is therefore the main determinant of the functional properties of the micro/nanocapsules and is considered the predominant performance indicator of a particular ingredient^17,18,28,29^. Commercial food grade encapsulants (wall materials) prevelant in the food industry are mostly polysaccharides, such as starch, cyclodextrins, alginates, chitosan, gum arabic, carboxymethyl cellulose, etc.^30,31^. These polysaccharides are usually derived from different plant, animal and marine sources^32–35^.

Encapsulants are generally the carriers of the targeted materials of importance and hence are never considered of any importance to the consumer in the food and pharmaceutical industry since they are only supposed to carry the material of importance to the targeted site of delivery, release it and get excreted out themselves. However, recently, there has been a lot on interest in carrier materials which themselves have some functional and/or nutraceutical property in themselves so that in addition to being carriers of the targeted materials can themselves also contribute towards enhancing the functionality of the food into which they are incorporated. Jackfruit seed is an underutilized source of starch, which is reported to possess all requisite characteristics and has a potential to be used as an alternate source of encapsulating/carrier material. The starch granules of the jackfruit seeds are similar to those present in cereals (in terms of a crystallinity standard), and its functional properties are reported to be better than many common starches being currently used by the food and pharmaceutical industry^36^. This makes jackfruit seed starch an interesting alternative carrier, which otherwise would have been discarded as waste.

Jackfruit seed contains about 13.50% protein 79.34% carbohydrates and 3.19% crude fiber^37^. Properties such as non-toxicity, gelling and thickening ability, stability to mechanical and thermal shear, binding capacity and acid resistivity makes Jack fruit seed starch (JSS) a widely applicable encapsulating agent in various food and pharmaceutical industries^38^. Soy protein isolate (SPI) in combination with starch is reported to be the most effective encapsulation material during spray drying^39,40^. Gum arabic, which acts as an emulsifying agent^41,42^ has been reportedly used for encapsulation of various pigments^34,41–43^, however, limited availability and high cost of production have been the limiting factors in the utilization of gum arabic in the food industries^44,45^. Similarly, NBRE-15, a novel emulsifier has been used effectively for the microencapsulation of anthocyanin^17,18^.

Of the many available methods of encapsulation, spray drying has been most commonly used by the food and pharmaceutical industry for microencapsulation simply because the process is relatively economical and readily available across different scales of commercial application^16,46,47^. Spray drying process involves an instantaneous simultaneous transfer of heat and mass from air to atomized droplet and vice versa which effectively helps in minimal deterioration of the inherent characteristics (physical as well as functional) of both the carrier as well as the bioactive target^48^.

In this study, SPI and JSS were used as a carrier material along with NBRE-15 as an emulsifier to encapsulate anthocyanin extracted from black carrot. Optimized combination of carrier material as well as the concentration of the emulsifier was determined by employing Box-Behnken Design (BBD) of Design of Experiments (DOE). Optimization was based on the monomeric anthocyanin content and antioxidant activity in the encapsulated microcapsules along with the encapsulation efficiency. Additionally, physical characterization of the microcapsules obtained using the optimized combination of encapsulants (SPI & JSS) as well as the emulsifier (NBRE-15) was carried out using particle size analysis and Scanning electron microscopy (SEM). Experiments in triplicate were also conducted using the optimized combination for validating the process of optimization.

## Materials and methods

### Materials

Black carrot (*Pusa asita*) harvested at the optimum maturity stage (90 to 100 days after planting) was sourced from the fields of the Division of Vegetable Science, Indian Agricultural Research Institute, New Delhi, India. The carrots were cleaned, washed and stored at 4–10 °C and 90–95% relative humidity for further use. Jackfruit seed starch was isolated from jackfruit seeds purchased from the local market in the laboratory using the method detailed below, Soy Protein Isolate (SPI) with >90% purity was purchased from the local market of New Delhi, India and verified for its purity (crude protein content, dry basis, N × 6.25) in the laboratory. NBRE-15 (an emulsifier) was obtained National Botanical Research Institute (NBRI), Lucknow, India. Other chemicals were purchased from Sigma Aldrich, India.

### Isolation of JSS

Dried jackfruit seeds (7.04% d.b.) were decorticated and milled using a hammer mill to obtain flour. Starch was then further extracted as described by Kar *et al*.^17^. The flour was sieved through the 150–170 µ mesh, a slurry was made by the addition of 75 mL of distilled water to 25 gram of flour. The slurry was centrifuged (Sigma 3–18 K, Germany) at 9000 rpm for 15 minutes at 20 °C, the sediment obtained was washed with 50% ethanol, after discarding the supernatant, and vacuum dried for 48 hours. Fat-free starch was finally obtained by defatting the residue using petroleum ether.

### Microencapsulation by spray drying

#### Preparation of core material

Black carrot stored at (4–10) °C was thawed to room temperature and anthocyanin was extracted as the method reported by Kar *et al*.^17^. Juice from carrot was extracted using a hydraulic press and filtered sequentially through a muslin cloth and Whatman No. 1 filter paper. 1% citric acid was added to it as a preservative and stored at 4 °C for further use as a core material.

#### Preparation of core and carrier material emulsion

A mixture of encapsulants (JSS, SPI & NBRE-15) weighed separately in the desired proportions were added to the carrot juice to obtain the desired overall TSS. The mixture was homogenized using an ultra-turrax homogenizer (IKA^®^T25 digital) thoroughly before they were fed into the pray dryer.

#### Spray drying

Spray drying (Sono Dry 1000, Sono-tek Corporation, USA) was used for encapsulation of the mixture prepared using anthocyanin extracted from black carrot as core material and JSS, SPI, and NBRE-15 as carriers. The inlet temperature of feed was set at 150 ± 2 °C and the outlet temperature for collecting the encapsulated material was set at 76 ± 2 °C. The feed rate was maintained at 2 mL/min throughout the process, with an aspirator speed of 79 rpm^17^. The encapsulated material was finally collected in amber-colored vials and stored in refrigerated condition for further physic-chemical analyses.

### Chemical characterization of the microcapsule

#### Monomeric anthocyanin content (MAC)

MAC was determined using a pH differential method^18^. Two buffers viz., pH 1.0 buffer (potassium chloride, 0.025 M) and pH 4.5 buffer (sodium acetate, 0.4 M) were used for the sample dilution and absorbance was measured at 520 nm and 700 nm by UV-Visible spectrophotometer (GENESYS 10 VIS, USA). Samples were taken in triplicates and the result was expressed in cyanidin-3-glucoside equivalents as:

1 $$MAC(mg/L)=\frac{A\times MW\times DF\times {10}^{3}}{\varepsilon \times 1}$$

Where;

2 $${\rm{A}}={\rm{Absorbance}}=({A}_{{\rm{520nm}}}-{A}_{700nm})pH\,1.0-({A}_{520nm}-{A}_{700nm})pH\,4.5$$

*MW* = Molecular weight = 449.2; *DF* = Dilution factor; 1 = path length in cm and *ε* = 26900 L mol^-1^ cm^-1^

#### Antioxidant activity

Antioxidant activity was determined using Cupric Reducing AOX capacity (CUPRAC) method^17^. To a test tube, 1 mL each of copper (II) chloride solution (10^−2^ M), neocuproine solution (7.5 × 10^−3^ M), and ammonium acetate buffer solution (pH 7) solution were added followed by water (1 mL) and AOX sample (0.1 mL) to make the final volume of 4.1 mL. After 1 hour, the absorbance was taken at 450 nm against a blank using a UV-Visible spectrophotometer (GENESYS 10 VIS, USA). The result was expressed by in 𝜇mol TE g^−1^.

#### Encapsulation efficiency

Encapsulation efficiency was calculated based on the content of anthocyanin in the juice before and after encapsulation. It was expressed in percentage as suggested in^17^.

3 $$Encapsulation\,efficiency( \% )=\frac{Total\,anthocyanin\,content\,in\,the\,feed}{Total\,anthocyanin\,content\,in\,the\,powder}\times 100$$

### Morphological characterization

#### Particle size analysis

The particle size distribution in the optimized encapsulated microcapsule was determined by Laser Scattering Particle Size Distribution Analyzer, LA-950 (Horiba Scientific Instruments, Japan). All samples were analyzed in triplicates.

#### Scanning electron microscope (SEM) analysis

Morphological characterization of encapsulated microcapsule obtained under optimized conditions was done by coating the microcapsule with gold/palladium to a thickness of 27 nm using an SC7620 mini sputter coater (Quorum Technologies Ltd, West Sussex, UK) followed by SEM analysis (Carl Zeiss, EVO/MA10, Germany). SEM was operated at 5000X magnification to analyze the nature of the surface of the microcapsules.

### Storage behavior kinetics

Degradation kinetics of encapsulated microcapsule was conducted at 37 °C in air-tight transparent and amber-colored vials with respect to the half-life of anthocyanin (t_1/2_), i.e. the time needed for its degradation by 50%^19^. Half-life was calculated by the following equation:

4 $${t}_{1/2}=-In0.5\times {k}^{-1}$$

Where,*In*(C_t_/C_0_) = −kt

Where C_t_ is the initial anthocyanin content, C_0_ is the anthocyanin content at the specific time and t is the time.

Monomaric anathocyanin content of the microcapsules stored in both the transparent as well as amber coloured vials were evaluated in triplicate at the beginning as well as after the lapse of 15 days interval upto 150 days of storage period.

### Experimental design and statistical analysis

Box-Behnken design (BBD) of the Design of Experiments (DOE) was used to optimize the effect of three independent variables i.e. TSS of feed to spray dryer – X_1_ (20, 25 and 30° Brix), ratio of SPI & JSS – X_2_ (1:1, 1:3 and 1:5) and concentration of NBRE-15 as an emulsifier – X_3_ (0.1, 0.2, and 0.3%) based on three dependent responses i.e. monomeric anthocyanin content – Y_1_, antioxidant activity – Y_2_ and encapsulation efficiency – Y_3_. The design resulted into 17 experimental combinations/runs detailed in Table 1. Effects of the independent variables (X_1_, X_2_, and X_3_) on any of the three responses Y was analysed using the following second-order polynomial equation using multiple regression

5 $${Y}_{n}={a}_{0}+{a}_{1}{X}_{1}+{a}_{2}{X}_{2}+{a}_{3}{X}_{3}+{a}_{11}{X}_{1}^{2}+{a}_{22}{X}_{2}^{2}+{a}_{33}{X}_{3}^{2}+{a}_{12}{X}_{1}{X}_{2}\,+\,{a}_{13}{X}_{1}{X}_{3}+{a}_{23}{X}_{2}{X}_{3}$$

Where *Y*_*n*_ represents one of the three response variables; X_1_, X_2_ and X_3_ coded values of the independent variables; *a*_*0*_ is a constant; *a*_*1*_, *a*_2_ and *a*_3_ are the coefficients of the linear effects; *a*_12_, *a*_*13*,_ and *a*_23_ are the coefficients of the interaction between the factors; and *a*_11_, *a*_*22*,_ and *a*_33_ are the coefficients of the quadratic effect. Analysis of variance (ANOVA) was performed to evaluate significant differences if any between the independent variables on the dependent variable using Design-Expert 8.0.7.1 (trial version).

**Table 1:** BBD design variables (TSS, ratio of SPI & JSS and NBRE-15) and responses for the optimization of encapsulating materials for the development of anthocyanin-rich powder.

Run	Independent Variables (X)			Responses (Y)		
	TSS (°Brix) X1	SPI:JSS (ratio) X2	NBRE-15 (%) X3	MAC (mg/100 g) Y1	AA (µmol Trolox/g) Y2	EE (%) Y3
1	20 (−1)	1:1 (−1)	0.2 (0)	2730.59	258.82	76.07
2	30 (1)	1:1 (−1)	0.2 (0)	2853.54	325.86	79.50
3	20 (−1)	1:5 (1)	0.2 (0)	2910.36	272.51	81.08
4	30 (1)	1:5 (1)	0.2 (0)	3062.72	345.81	85.33
5	20 (−1)	1:3 (0)	0.1 (−1)	2670.65	256.48	74.40
6	30 (1)	1:3 (0)	0.1 (−1)	2787.34	308.27	77.65
7	20 (−1)	1:3 (0)	0.3 (1)	2730.86	275.82	76.08
8	30 (1)	1:3 (0)	0.3 (1)	2805.63	316.16	78.17
9	25 (0)	1:1 (−1)	0.1 (−1)	2985.23	328.15	83.17
10	25 (0)	1:5 (1)	0.1 (−1)	3103.30	354.47	86.46
11	25 (0)	1:1 (−1)	0.3 (1)	3030.27	348.90	84.43
12	25 (0)	1:5 (1)	0.3 (1)	3250.86	365.26	90.57
13	25 (0)	1:3 (0)	0.2 (0)	3030.48	348.91	83.28
14	25 (0)	1:3 (0)	0.2 (0)	3010.36	342.36	82.73
15	25 (0)	1:3 (0)	0.2 (0)	3016.35	347.45	82.89
16	25 (0)	1:3 (0)	0.2 (0)	3078.29	352.56	84.62
17	25 (0)	1:3 (0)	0.2 (0)	3092.36	338.12	85.02

**Note:** MAC – monomeric anthocyanin content; AA – antioxidant activity; EE – encapsulation efficiency. *Values in the parenthesis mentions coded values of BBD of DOE*.

Response surfaces responses and contour plots were plotted to have a greater understanding of the relationship between the variables and the responses of the study.

### Compliance with ethics requirement

It is certified that:

- The manuscript has not been submitted to more than one journal for simultaneous consideration.
- The manuscript has not been published previously (partly or in full).
- A single study is not split up into several parts to increase the number of submissions and submitted to various journals or one journal over time.
- No data have been fabricated or manipulated (including images) to support our conclusions
- No data, text, or theories by others are presented as if they were the author’s own (“plagiarism”). Proper acknowledgments to other works have been given, quotation marks are used for verbatim copying of material, and permissions are secured for copyrighted material.
- Consent to submit has been received explicitly from all co-authors, as well as from the responsible authorities - tacitly or explicitly - at the institute/organization where the work has been carried out before the work is submitted.
- Authors whose names appear on the submission have contributed sufficiently to the scientific work and therefore share collective responsibility and accountability for the results.

### Ethical approval

#### Informed consent

This article does not contain any studies with either animals or human participants performed by any of the authors.

## Results and Discussion

The details of the variables used in the 17 designed experimental runs as well as the responses obtained have been presented in Table 1.

### Effect of independent variables on monomeric anthocyanin content (MAC)

**Figure 1:**
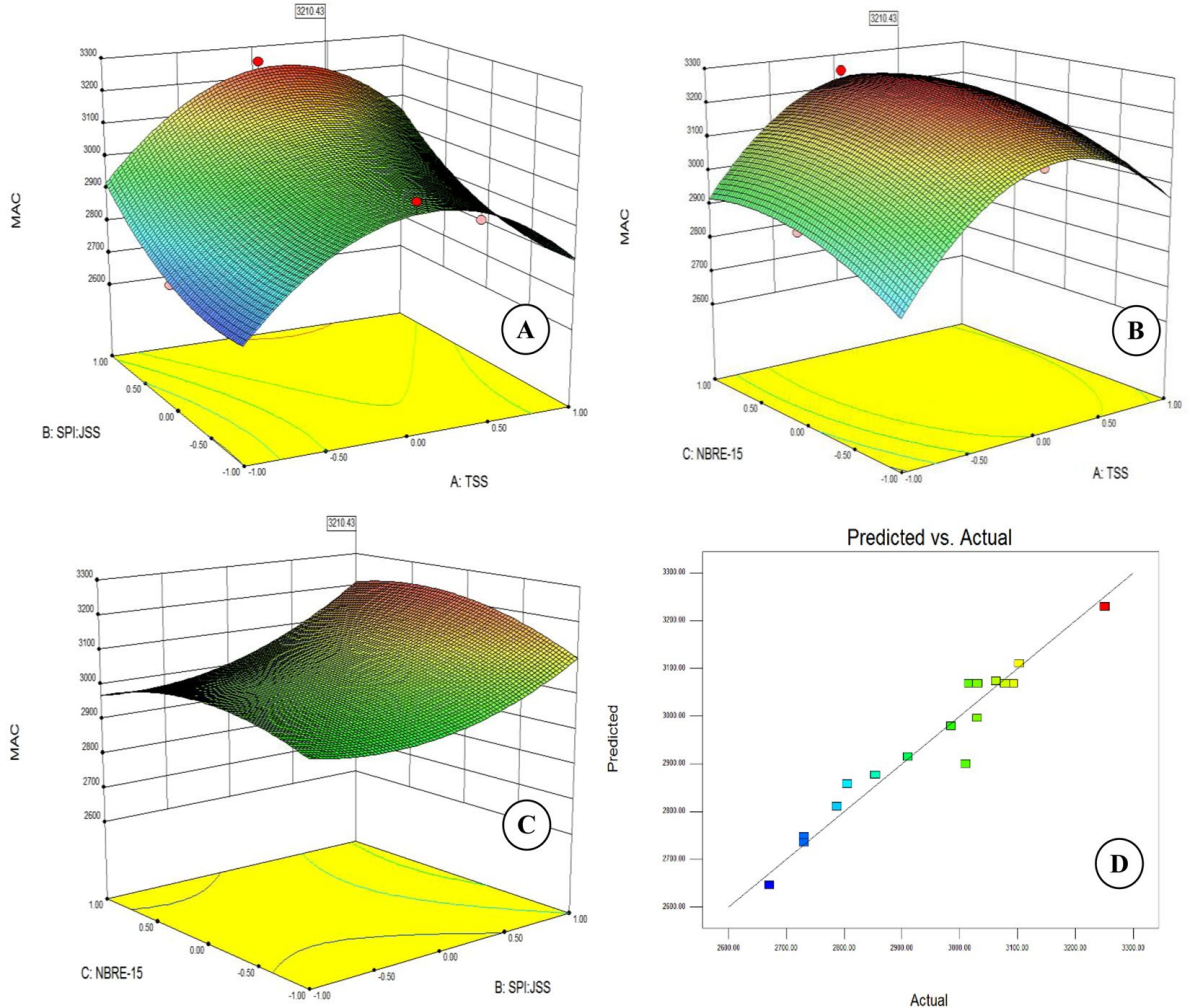
Surface plots showing effect of independent variables on the anthocyanin content. (**A**) TSS & ratio of SPI: JSS; (**B**): TSS and NBRE-15; (**C**): ratio of SPI: JSS & NBRE-15; and (**D**): predicted vs actual plot for MAC.

Table 1 clearly indicates that MAC varied between a minimum value 2670.65 mg/100 g to 3250.86 mg/100 g compared to the 3389.26 mg/100 g. Response surface plot (Fig. 1) clearly indicated that MAC increased with the increase in the ratio of SPI:JSS at any given combination of TSS & NBRE-15. This clearly indicates that increase in the content of JSS in the encapsulant matrix increases the MAC which can be because of the presence of polysaccharides such as amylose and amylopectin in JSS, which possesses better binding properties^38^. Robert *et al*.^38^ also found that the increasing SPI content during encapsulation of anthocyanin from pomegranate (*Punica granatum*) diminished the anthocyanin content.

Increase in TSS from 20 to 25° Brix results in higher MAC at any given ratio of SPI:JSS because of increased availability of encapsulant which protects the anthocyanins from degradation^49^. However, further increase beyond 25° Brix resulted in a sharp decrease in MAC which maybe due to the dilution of MAC in the feed mixture^17^. This can be attributed to the t at higher TSS leading to a higher degree of encapsulation. This is clearly corroborated with the simultaneous changes in the encapsulation efficiencies in accordance with the MAC content. Similar trend was also observed with the change in the TSS content at any given concentration of NBRE-15. Increase in the concentration of NBRE-15 however, did not seem to have any significant effect on MAC with the change of either of TSS or SPI:JSS ratio. Value of regression coefficients obtained (Eq. 6) also clearly indicates the ratio of SPI:JSS as the most dominant factor affecting MAC followed by TSS & concentration of NBRE-15. Results indicate very little effect of the emulsifier i.e. NBRE-15 on the MAC.

Regression analysis for the interaction effects among the independent variables on the monomeric anthocyanin content indicated a positive effect of the interactions between TSS & SPI:JSS and SPI:JSS & NBRE-15, whereas the interaction between TSS and NBRE-15 found to have a negative effect. The regression coefficients obtained for predicting the monomeric anthocyanin content is as follows:

6 $$\begin{array}{rcl}Y & = & 3045.56+58.35{X}_{1}+90.95{X}_{2}+33.88{X}_{3}-250.03{X}_{1}^{2}\\  &  & +93.76{X}_{2}^{2}-46.91{X}_{3}^{2}+7.35{X}_{1}{X}_{2}-10.48{X}_{1}{X}_{3}+25.63{X}_{2}{X}_{3}\end{array}$$

A diagnostic plot between predicted (obtained using the regression co-efficients) and actual (experimentally obtained) values was drawn to assess the suitability of the second order polynomial used for the regression analysis (Fig. 1D). A linear relationship between the observed and the predicted values clearly indicated the suitability of the second order polynomial in accurately describing the variability in MAC obtained using differen combinations of the independent variables.

### Effect of independent variables on antioxidant activity (AA)

**Figure 2:**
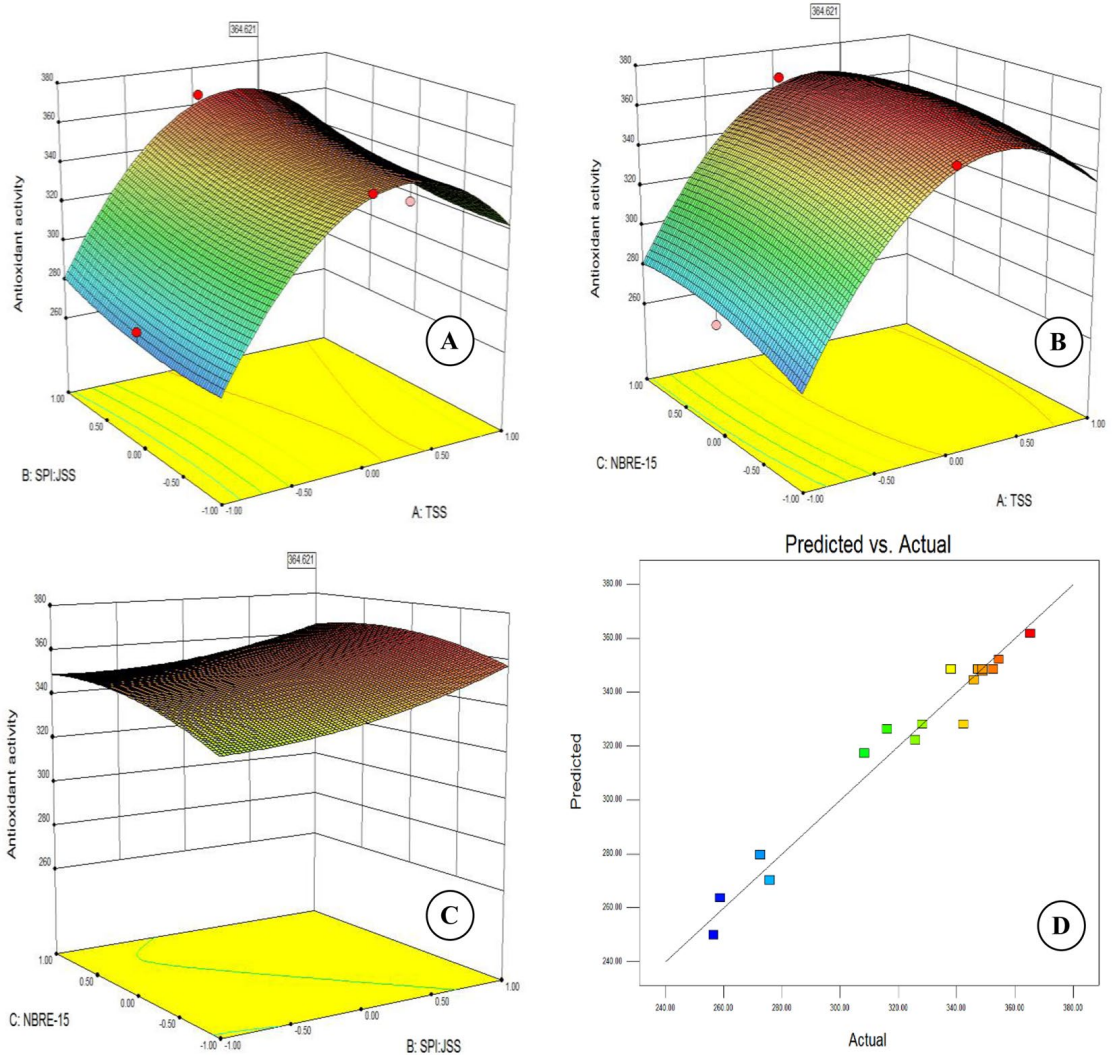
Surface plots showing effect of independent variables on the antioxidant activity (**A**) TSS & ratio of SPI: JSS; (**B**): TSS and NBRE-15; (**C**): ratio of SPI: JSS & NBRE-15; and (**D**): predicted vs actual plot for antioxidant activity.

The antioxidant activity of microcapsules ranged between 256.48 to 365.26 µmol Trolox/g dry matters (Table 1). Response plots (Fig. 2), clearly indicates a sharp increase in the antioxidant activity at any given ratio of SPI:JSS as well as NBRE-15 concentration upto about 27° Brix. This may have been attributed to the increased availability of carrier material to substantially and effectively encapsulate the available anthocyanin in the feed mixture. With the increase in the TSS beyond 27° Brix however, leads to a sharp decline in the anthocyanin activity. This is attributed to the excessive availability of carrier which had no available anthocyanin to encapsulate i.e. there is simply additional carrier in the final product which in turn significantly reduces the concentration of anthocyanin. The interaction between the ratio of SPI:JSS and the NBRE-15 however did not seem to have any effect on the antioxidant activity i.e. neither change in ratio of SPI:JSS at any given concentration of NBRE-15 nor the change in concentration od NBRE-15 at any given ratio of SPI:JSS led to any significant change in the antioxidant activity of the microcapsules. This is contrary to the reported literature that emulsifiers themselves having antioxidant properties contribute towards the enhancement of antioxidant activity^17,50^.

Regression coefficients of the second order polynomial (Eq. 7) used to describe the variability in the antioxidant activity clearly indicates the predominance of TSS (X_1_) compared to the other two independent variables i.e. SPI:JSS (X_2_) & concentration of NBRE-15 (X_3_). Of the interaction effects, the interaction between TSS & SPI:JSS (X_1_×_2_) has a positive effect on the antioxidant activity. The other two negatively effect the anthocyanin activity.

7 $$\begin{array}{rcl}Y & = & 345.88+29.06{X}_{1}+9.54{X}_{2}+7.35{X}_{3}-52.57{X}_{1}^{2}+7.44{X}_{2}^{2}-4.12{X}_{3}^{2}\\  &  & +1.56{X}_{1}{X}_{2}-2.86{X}_{1}{X}_{3}-2.49{X}_{2}{X}_{3}\end{array}$$

As in case of the MAC, the diagnostic plot (Fig. 2D) exhibited the linear relationship with intercept as zero, between predicted and actual antioxidant activity indicating the suitability of the model in effectively describing the variability in the antioxidant activity obtained during the experimentation.

### Effect of independent variables on encapsulation efficiency

**Figure 3:**
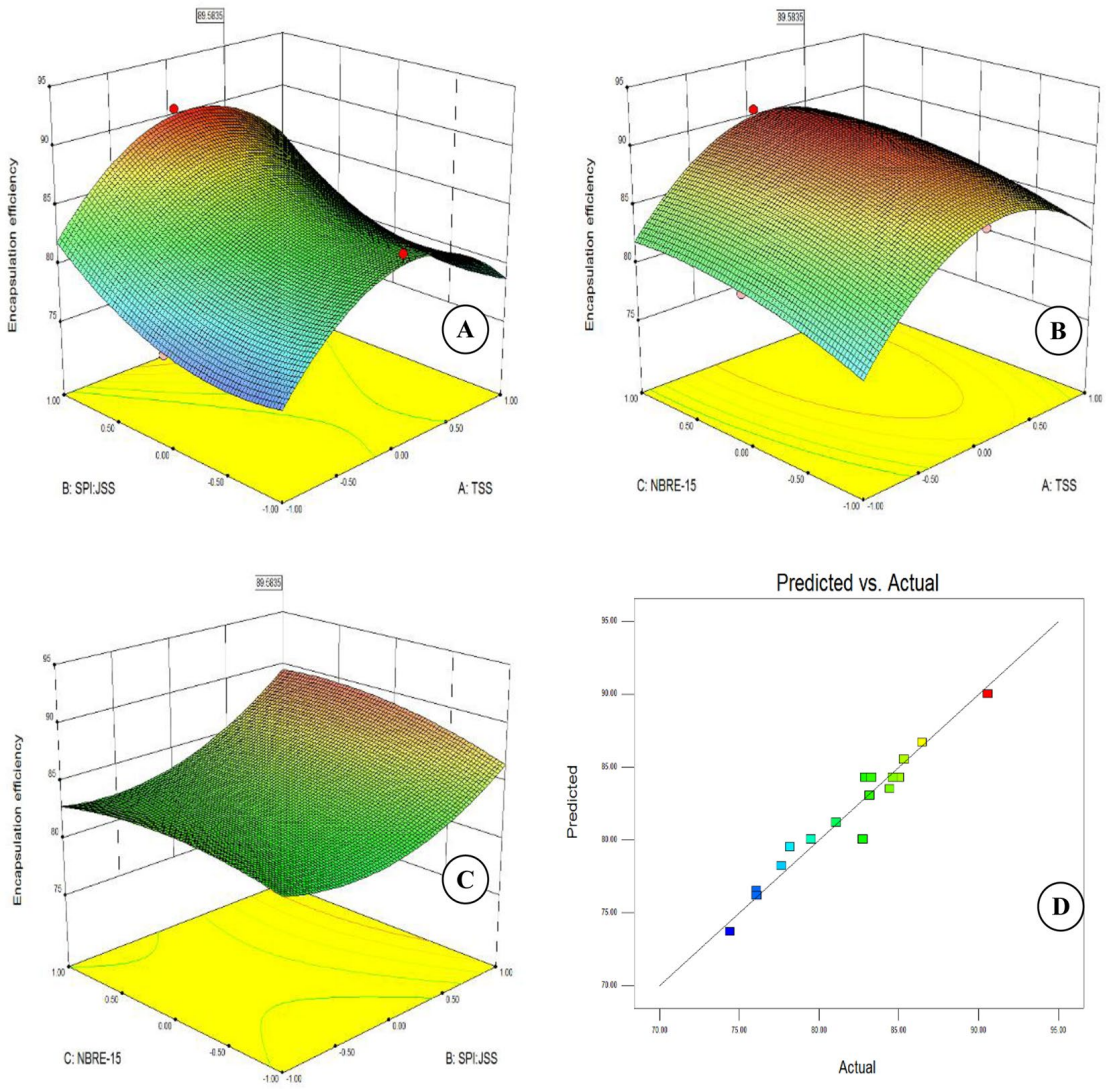
Surface plots showing effect of independent variables on the encapsulation efficiency (**A**): TSS & ratio of SPI: JSS; (**B**): TSS and NBRE-15; (**C**): ratio of SPI: JSS & NBRE-15; and (**D**): predicted vs actual plot for encapsulation efficiency.

Encapsulation efficiency is an important indicator for microencapsulated particles that refers to the potential of the wall material to encapsulate or hold the core material inside the microcapsule. It varied between 76.07 to 90.57% with the use of different experimental combinations of the independent variable. With the increase in the ratio of the SPI:JSS at any given TSS from 1:3 to 1:5 the encapsulation efficiency does not show any significant change (Fig. 3A), whereas increase beyond 1:3 upto 1:5 significantly enhances encapsulation efficiency at any given TSS. This result is in agreement with our previous finding which showed that the higher the ratio of the starch with whey protein led to the higher encapsulation efficiency of the anthocyanin^17^. Therefore, the utilization of JSS in the encapsulation had a higher potential to encapsulate the anthocyanins over SPI. On the other hand at given ratio of SPI:JSS as well as concentration of NBRE-15, increase in TSS from 20 to 27°Brix significantly increased the encapsulation efficiency which also reduced significantly on further increase of TSS between 27 and 30°Brix (Fig. 3A,B). At any given TSS, increase in concentration of emulsifier (NBRE-15) generally leads to increase in encapsulation efficiency. The increase however is more between 0.1% and 0.2% compared to those between 0.2 and 0.3%.

At lower ratio of SPI:JSS i.e. between 1:1 to 1:3, concentration of NBRE-15 has a non-significant effect on the encapsulation efficiency. However, increase beyond 1:3 upto 1:5, increase in NBRE-15 concentration upto 0.25% has a positive effect on the encapsulation efficiency. Further increase in concentration of NBRE-15 beyond 0.25% has no effect whatsoever on the encapsulation efficiency (Fig. 3C). The encapsulation efficiency of the anthocyanin at 0.1% concentration of NBRE-15 was significantly (p < 0.05) lower than 0.2% and 0.3%. This could be attributed to the insufficient or lack of emulsifying properties and lower film-forming capacity of the NBRE-15 at lower concentration.

Coefficients of the second order polynomial (Eq. 8) obtained during the regression analysis indicates the ratio of SPI:JSS (X_2_) having the maximum effect on the encapsulation efficiency followed TSS (X_1_) and NBRE-15 (X_3_).

8 $$\begin{array}{rcl}Y & = & 83.71+1.63{X}_{1}+2.53{X}_{2}+0.94{X}_{3}-6.39{X}_{1}^{2}+3.18{X}_{2}^{2}\\  &  & -0.73{X}_{3}^{2}+0.21{X}_{1}{X}_{2}-0.29{X}_{1}{X}_{3}+0.71{X}_{2}{X}_{3}\end{array}$$

A linear diagnostic plot (Fig. 3D) between predicted and actual values of encapsulation efficiency indicates the suitability of the second order polynomial in describing adequately the variability in the encapsulation efficiencies achieved using different combinations of the independent variables.

### Optimization of combination of independent variables vis-à-vis the responses

ANOVA was performed to evaluate the significance of coefficients of the quadratic polynomial models and the lack of fit for all fitted models was found to be non significant (p > 0.05) whereas model for all the responses were found to be significant (p ˂ 0.05). An attempt was therefore made to optimize the combination of the independent variables i.e. TSS, ratio of SPI:JSS and NBRE-15 by targeting the variables and the responses both up to maximum goal with high importance. The constraints for the optimization are presented in in Table 2. Analysis resulted in a combination of X1 = 0.46 i.e. 27.3 °Brix TSS; X2 = 1 i.e. ratio of 1:5:: SPI:JSS; and X3 = 1 i.e. NBRE-15 concentration of 0.3%. The optimum combinations were predicted to have MAC of 3210.43 mg/100 g dry matter, antioxidant capacity of 364.62 µmol Trolox/g dry matter and encapsulation efficiency of 89.58%. The optima so obtained had a desirability of 92.6% (Table 3).

**Table 2:** Constraints and importance for the optimization of independent for maximizing the desired responses.

Name	Goal	Lower Limit	Upper Limit	Lower Weight	Upper Weight	Importance
A:TSS	maximize	−1	1	1	1	3
B:S:JSS	maximize	−1	1	1	1	3
C:NBRE-15	maximize	−1	1	1	1	3
Anthocyanin content	maximize	2670.65	3250.86	1	1	3
Antioxidant activity	maximize	256.48	365.26	1	1	3
Encapsulation efficiency	maximize	74.40	90.57	1	1	3

**Table 3:** Desirability of different combinations of independent variables and their predicted responses.

No.	TSS	SPI:JSS	NBRE-15	MAC	AA	EE	Desirability
1	0.46	1.00	1.00	3210.43	364.621	89.5835	0.926
2	0.44	1.00	1.00	3212.42	364.844	89.6333	0.926
3	0.47	1.00	1.00	3208.71	364.43	89.5389	0.926
4	0.45	1.00	0.99	3211.46	364.743	89.5999	0.926
5	0.48	1.00	1.00	3205.41	364.073	89.4507	0.925
6	0.39	1.00	1.00	3219.24	365.528	89.8034	0.925
7	0.52	1.00	0.97	3201.28	363.482	89.3231	0.924
8	0.47	0.99	1.00	3206.26	364.273	89.4583	0.923
9	0.52	1.00	0.91	3206.74	364.173	89.4002	0.922
10	0.26	1.00	1.00	3230.58	366.039	90.0819	0.916
11	0.59	1.00	0.74	3202.51	363.577	89.141	0.909
12	0.85	1.00	1.00	3113.07	349.762	87.1199	0.883

In order to validate the optima, three experimental runs were conducted using the obtained combinations of 27.3°Brix, 1:5:: SPI:JSS; and 0.3% NBRE-15 and the responses recorded for each of the three experimental runs in triplicate. The results are presented in Table 4. Close proximity of the observed and predicted responses establishes the validity of the optimization process.

**Table 4:** Experimental and predicted values of responses obtained using microcapsules produced using optimum conditions of independent variables i.e. TSS 27°Brix, 1:5:: SPI:JSS; and 0.3% NBRE-15.

MAC (mg/100 g dry matter)		AA (µmol Trolox/g dry matter)		EE (%)	
Observed	Predicted	Observed	Predicted	Observed	Predicted
3248.48 ± 32.16	3210.43	366.12 ± 2.47	364.62	90.62 ± 1.22	89.58

All observed values presented as mean ± SD.

### Morphological characterization of microcapsules obtained using optimized combination of TSS, ratio of SPI:JSS and concentration of NBRE-15

#### Particle size distribution

**Figure 4:**
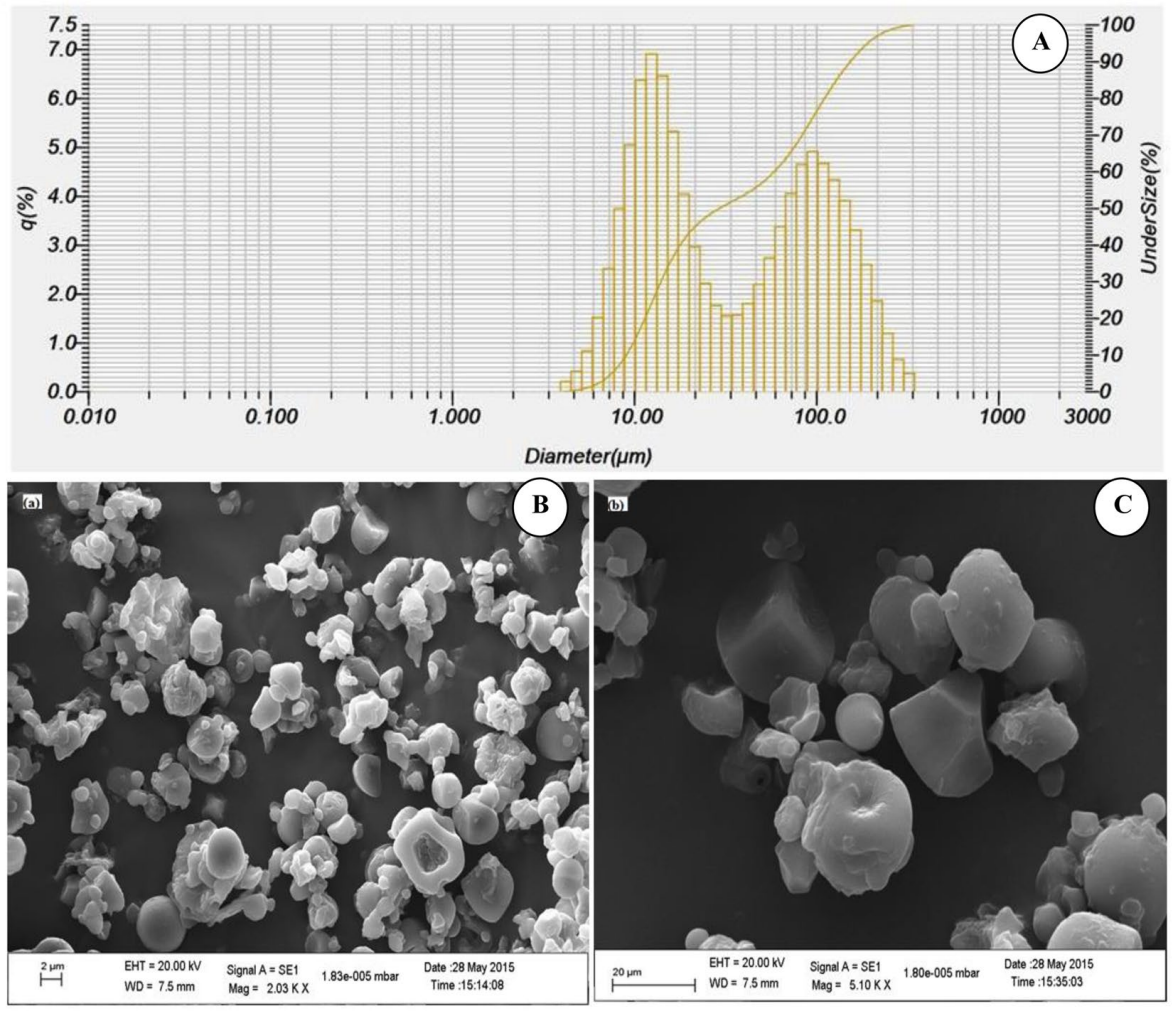
Morphological characterization of microcapsules obtained using optimum combinations of TSS (27°Brix), SPI:JSS (1:5) and NBRE-15 (0.3%) (**A**): Particle size distribution; (**B**): SEM image with 2000X magnification; and (**C**): SEM image with 5000X magnification.

The particle size distribution for the microcapsules is shown in Fig. 4A. 10% of the microcapsules i.e. D_10_ was found to be below 8.99 µm, 50% of the microcapsules i.e. D_50_ was found to be below 29.21 µm and 90% of the microcapsules i.e. D_90_ was found to be below 152.23 µm. Average particle size of the microcapsules i.e. D_mean_ was found to be 60.97 µm.

#### Scanning electron microscope (SEM) analysis

Figure 4B,C presents SEM image of the microencapsules containing anthocyanin at magnifications of 2000X and 5000X respectively. Figures clearly indicate an oval to slightly cuboidal shape of the microcapsules having a smooth surface with minimum surface cracks. Smooth surface of microcapsules without cracks indicates the suitability of safe encapsulation of the targeted compound^31^. Some particle agglomeration is also seen which may have been due to the inherent stickiness of the different components of the complex encapsulating matrix^17^.

### Storage stability of the encapsulated anthocyanin

**Figure 5:**
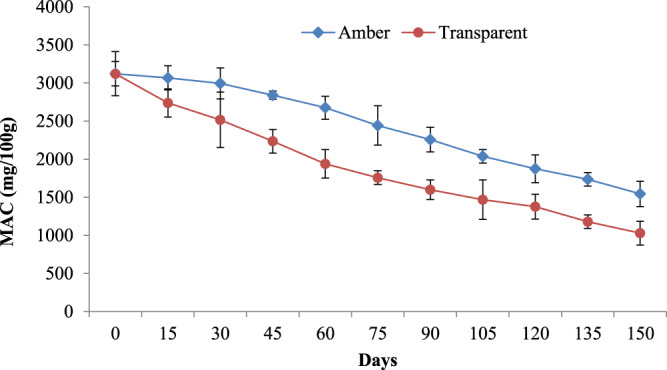
Degradation Kinetics of monomeric anthocyanin content encapsulated using the optimized combinations of 27°Brix TSS of spray feed, 1:5 ratio of the SPI:JSS and 0.3% NBRE-15.

Degradation kinetics of the anthocyanin encapsulated within the microcapsules was studied during its storage in transparent as well as amber coloured air tight vials at 37 °C. Results of the same is presented in Fig. 5. As expected the rate of degradation of anthocyanin was found to significantly higher in the microcapsules stored in transparent vials compared to those stored in amber coloured vials. It is clearly because of the restriction of contact of anthocyanin with light in amber coloured vials as is corroborated from the study on the storage stability of encapsulated anthocyanin by Mahdavi *et al*.^19^.

The half-life i.e. 50% retention of anthocyanin in the microcapsules stored under amber-colored vial was found to be 63 days compared to only 35 days for those stored in transparent vials. These results are many times higher than those obtained during the storage of anthocyanin juice at similar and accelerated temperatures^51,52^. Janna *et al*.^53^ also reported significant decline i.e. degradation of upto 50% of anthocyanins exposed to light at 25 °C. Mahdavee *et al*.^19^ reported the half-life of encapsulated anthocyanin in maltodextrin and gum arabic from saffron petals as 10 weeks with 32% of total anthocyanin retention at 35 °C.

## Conclusion

The optimization process vis-à-vis the effect of independent variables of the dependent ones indicated that the optimum combinations of TSS of feed, ratio of SPI & JSS and concentration of emulsifier i.e. NBRI-15 were 27.3°Brix, 1:5 & 0.3% respectively. Encapsulation using these optimum conditions resulted in microcapsules having 3215.59 mg/100 g MAC, 365.26 µmol Trolox/g antioxidant activity and an encapsulation efficiency of 89.71%. Particle size analysis of the microcapsules obtained using the optimum combinations has an average size of 60.97 µm. Morphological evaluation using SEM indicated the microcapsules obtained were oval and cuboidal in shape with smooth surfaces. Half-life of the encapsulated anthocyanin when stored under air tight amber containers at 37 °C was found to be 63 days. The study clearly justifies the suitability of use of jackfruit seed starch (JSS) for use as an encapsulant for bioactive compounds in the food industry.
